# The repertoire and levels of secondary metabolites in microbial cocultures depend on the inoculation ratio: a case study involving *Aspergillus terreus* and *Streptomyces rimosus*

**DOI:** 10.1007/s10529-024-03500-4

**Published:** 2024-06-06

**Authors:** Tomasz Boruta, Grzegorz Englart, Martyna Foryś, Weronika Pawlikowska

**Affiliations:** Department of Bioprocess Engineering, Faculty of Process and Environmental Engineering, Lodz University of Technology, ul. Wólczańska 213, 93-005 Lodz, Poland

**Keywords:** *Aspergillus terreus*, Cocultures, Secondary metabolites, *Streptomyces rimosus*, Inoculation ratio

## Abstract

**Objective:**

The aim of this study was to determine the influence of the inoculation volume ratio on the production of secondary metabolites in submerged cocultures of *Aspergillus terreus* and *Streptomyces rimosus*.

**Results:**

The shake flask cocultures were initiated by using 23 inoculum variants that included different volumes of *A. terreus* and *S. rimosus* precultures. In addition, the axenic controls were propagated in parallel with the cocultures. UPLC‒MS analysis revealed the presence of 15 secondary metabolites, 12 of which were found both in the “*A. terreus* vs. *S. rimosus*” cocultures and axenic cultures of either *A. terreus* or *S. rimosus*. The production of the remaining 3 molecules was recorded solely in the cocultures. The repertoire and quantity of secondary metabolites were evidently dependent on the inoculation ratio. It was also noted that detecting filamentous structures resembling typical morphological forms of a given species was insufficient to predict the presence of a given metabolite.

**Conclusions:**

The modification of the inoculation ratio is an effective strategy for awakening and enhancing the production of secondary metabolites that are not biosynthesized under axenic conditions.

## Introduction

The interest in microbial cocultures has been growing continuously in recent years, in concert with the achievements made with regard to the characterization of the intestinal microbiome, the construction of artificial microbial communities and the understanding of ecological interactions between microorganisms in the natural environment (Burz et al. 2023; Fan and Pedersen 2021; Thomas et al. 2024). The currently observed “paradigm shift” in microbiological sciences involves, among other factors, progressing from the conventional approach of investigating microbial monocultures (axenic cultures) toward employing cocultures of two or more species (or strains) that are free to interact with each other (Nai and Meyer 2018). The nature of such interactions may be chemical (i.e., via the synthesized molecules) and/or physical (i.e., through direct contact between the cells), depending on the cultivation vessel design and the presence or absence of barriers (e.g., membranes) that are impenetrable for cells (Kapoore et al. 2022). The motivation behind establishing microbial cocultures varies greatly depending on the research area and experimental objectives. Among the applications with proven effectiveness, the coculture-based induction of biosynthetic pathways is of particular relevance for the pharmaceutical industry in the context of drug discovery pipelines (Peng et al. 2021; Wu et al. 2015). While there are countless published accounts reporting the awakening of silent metabolic pathways through cocultivation, they typically focus on the discovery of novel molecules, often of remarkable structural complexity (Arora et al. 2020), rather than on the underlying methodological intricacies. Underscoring the successful outcomes of cocultures is perfectly understandable, however, the challenges and difficulties encountered in relation to the experimental design are still insufficiently addressed in the literature. Importantly, there are multiple bioprocess-related issues inherently associated with microbial cocultivation, which, especially at the early stages of research projects, require much attention, time, and effort. Among them, achieving a state in which two distinct species coexist under laboratory conditions without outgrowing one another is a particular difficulty (Gutiérrez Mena et al. 2022; Ibrahim et al. 2021; Schlembach et al. 2021).

In contrast to the microbial interdependencies that have shaped natural environments throughout evolutionary history, physical and chemical interactions occurring over the course of laboratory cocultivation are typically short-term and follow the “winner-takes-all” scenario. In other words, the slower-growing, less “aggressive” strain is likely to be outperformed and eliminated from coculture by the dominant, faster-growing microbe (Boruta et al. 2020; Martinez et al. 2022). This is highly problematic, especially if the target products are expected to be biosynthesized by the former microorganism. Among the strategies devised to address this issue in the coculture context, the adjustment of the inoculation ratio is often chosen as a first-resort approach (Akdemir et al. 2019; Carlson et al. 2015; Gao et al. 2020; Jones et al. 2016; Karuppiah et al. 2019; Li et al. 2018; Shin et al. 2018; Yuan et al. 2020). Depending on the types of microorganisms and research goals, one may consider various inoculum characteristics, such as the relative number of cells at the time of coculture inoculation, the relative biomass concentration, or the optical density of the seed culture. Arguably, the most convenient method, which does not require any analytical effort at the inoculum development stage, is based on the fine-tuning of the inoculation volume ratio (Cho and Kim 2012; Ezaki et al. 1992; Jiang et al. 2021; Onaka et al. 2011). The volumes of the precultures used for coculture initiation (with each preculture representing one of the strains) can be optimized to reach the desired effect in terms of microbial population growth. Such studies are typically focused on the biosynthesis of target molecules, while the landscape of other products generated over the course of coculturing remains uncharacterized. In biotechnology, the formation of biosynthetic byproducts is recognized as a serious issue at the stage of downstream processing, when the target chemical is isolated and purified. For example, in bioprocesses involving the fungus *Aspergillus terreus,* the production of lovastatin (a cholesterol-lowering statin drug) is commonly accompanied by the formation of other molecules, most notably (+)-geodin (Calam et al. 1947). Similarly, during the production of oxytetracycline (a widely prescribed antibiotic) in the cultures of the actinomycete *Streptomyces rimosus,* the byproduct 2-acetyl-2-decarboxamido-oxytetracycline (ADOTC) is often detected (Hochstein et al. 1960), and in addition, the species has numerous other secondary metabolites in its catalog (Slemc et al. 2022). While the landscape of secondary metabolites of filamentous microorganisms has been thoroughly investigated in the context of bioprocess-related aspects of axenic cultures, this topic remains underrepresented in the field of coculture research. Currently, there is still a need to investigate (both qualitatively and quantitatively) the production of secondary metabolites in cocultures of filamentous microbial strains in relation to the inoculation volume ratio. Here, submerged cocultures of *A. terreus* and *S. rimosus* were examined as a model system involving two microorganisms that provide industrially important secondary metabolites (i.e., lovastatin and oxytetracycline, respectively) but are also capable of producing a wide array of other structurally diverse secondary metabolites, which, depending on the context, can be considered either unwanted byproducts or potentially relevant bioactive molecules. Moreover, the “*A. terreus* vs. *S. rimosus*” cocultures were previously shown to yield secondary metabolites that were absent from the corresponding axenic variants, including products possibly originating from the biosynthetic family of rimocidins (Boruta et al. 2021).

The aim of this study was to characterize the influence of the inoculation volume ratio on the repertoire of secondary metabolites produced by *A. terreus* and *S. rimosus* in submerged cocultures.

## Materials and methods

### Strains

The strains *Aspergillus terreus* ATCC 20542 and *Streptomyces rimosus* ATCC 10970, both of which were purchased from the American Type Culture Collection, were used in the study. The strains were maintained on agar slants at 4 °C.

### Media

The following liquid media were used throughout the study (both for the precultures and experimental variants): lactose, 20 g L^−1^; glucose, 20 g L^−1^; yeast extract, 5 g L^−1^; KH_2_PO_4_, 1.51 g L^−1^; NaCl, 0.4 g L^−1^; MgSO_4_ ∙ 7 H_2_O, 0.52 g L^−1^; ZnSO_4_ ∙ 7 H_2_O, 1 mg L^−1^; Fe(NO)_3_ ∙ 9 H_2_O, 2 mg L^−1^; biotin, 0.04 mg L^−1^; and trace element solution, 1 mL L^−1^. The following trace element solution was used: H_3_BO_3_, 65 mg L^−1^; MnSO_4_ ∙ 7 H_2_O, 43 mg L^−1^; CuSO_4_ ∙ 5 H_2_O, 250 mg L^−1^; and Na_2_MoO_4_ ∙ 2 H_2_O, 50 mg L^−1^. The pH of the liquid medium was set to 6.5 by using a 1 M solution of NaOH.

The composition of the sporulation medium for *A. terreus* was as follows: casein peptone, 5 g L^−1^; malt extract, 20 g L^−1^; and agar, 30 g/L. Commercially available ISP Medium 2 (BD, USA) was used for the sporulation of *S. rimosus*.

All media were sterilized by autoclaving at 121 °C for 20 min.

### Cultivation

The spores of both species were obtained via cultivation on agar slants for 10 days at 28 °C. The precultures of *A. terreus* and *S. rimosus* were started by transferring the spores of a given species from agar slants into 200 mL of sterile liquid medium by using a disposable 1 mL plastic pipette to achieve (1,0 ± 0,1) ∙ 10^9^ spores per liter. The precultures were propagated (as axenic cultures) for 24 h. Then, a total inoculum volume of 20 mL (containing the specified volumes of *A. terreus* and *S. rimosus* precultures) was transferred to 200 mL of production medium to achieve a total working volume of 220 mL for each experimental variant. The variants (specified in Table 1) differed with respect to the volumes of *A. terreus* and *S. rimosus* precultures included in the total volume (i.e., 20 mL) of inoculum. The set of variants (Table 1) comprised 23 “*A. terreus* vs. *S. rimosus*” cocultures and two axenic cultures (one of *A. terreus* and one of *S. rimosus*).

**Table 1:** Preculture volumes corresponding to the investigated experimental variants

Experimental variant	Volume of preculture, mL	
	*A. terreus*	*S. rimosus*
Axenic culture of *S. rimosus*	0	20
Coculture #1	0.25	19.75
Coculture #2	0.5	19.5
Coculture #3	1	19
Coculture #4	2	18
Coculture #5	3	17
Coculture #6	4	16
Coculture #7	5	15
Coculture #8	6	14
Coculture #9	7	13
Coculture #10	8	12
Coculture #11	9	11
Coculture #12	10	10
Coculture #13	11	9
Coculture #14	12	8
Coculture #15	13	7
Coculture #16	14	6
Coculture #17	15	5
Coculture #18	16	4
Coculture #19	17	3
Coculture #20	18	2
Coculture #21	19	1
Coculture #22	19.5	0.5
Coculture #23	19.75	0.25
Axenic culture of *A. terreus*	20	0

Liquid cultivation was performed in flat-bottom shake flasks (total volume: 500 mL, working volume: 220 mL). An Innova S44i (Eppendorf, Germany) laboratory shaker was used for all shake flask cultivations. The temperature and shaking speed were set to 28 °C and 120 min^−1^, respectively, and kept constant throughout the bioprocess. The cultivation times were 24 and 168 h for the precultures and experimental variants, respectively.

### Analysis

Secondary metabolites were analyzed in liquid samples after biomass removal via filtration. An AQUITY-UPLC system equipped with a BEH Shield RP18 (reversed-phase) chromatographic column (2.1 mm × 100 mm × 1.7 μm) coupled with a SYNAPT G2 high-resolution mass spectrometer (Waters, USA) was used throughout the course of the study to identify and quantify the metabolites produced by *A. terreus* and *S. rimosus*.

The following water:acetonitrile gradient (both eluents contained formic acid at 0.1%) was used for elution: 0 min, 100:0 (v/v); 2.5 min, 80:20 (v/v); 5.5 min, 70:30 (v/v); 7.5 min, 60:40 (v/v); and 14.0 min, 40:60 (v/v).

MS analysis was performed in ESI−mode. The parameters were: capillary voltage, 3 kV; sampling cone, 40 V; extraction cone, 4 V; temperature of the source, 120 °C; and temperature of desolvation, 200 °C. The identification of secondary metabolites was performed with the use of The Natural Product Atlas (van Santen et al. 2022) and literature records on the basis of *m/z* values. There were two conditions that had to be satisfied before suggesting a metabolite name. Firstly, the differences between the experimental and calculated *m/z* values that corresponded to the [M–H]^−^ ions were always smaller than 0.01. Secondly, all suggested metabolites were previously associated with the biosynthetic activity of *A. terreus* and *S. rimosus*. The identities of mevinolinic acid (acidic form of lovastatin), oxytetracycline, (+)-geodin, and butyrolactone I were confirmed by using authentic standards. The standards of mevinolinic acid, oxytetracycline, and (+)-geodin were purchased from Sigma-Aldrich (USA). The standard of butyrolactone I was obtained from Enzo Life Sciences (USA). The remaining metabolites were putatively annotated when the *m/z* error was not greater than 0.01, and the suggested metabolite name was assigned to the species *S.* *rimosus* or *A. terreus* in previous literature reports. The quantification procedure developed in TargetLynx software (Waters, USA) was employed to determine the peak areas corresponding to the identified [M–H]^−^ ions. The concentrations of the industrially important metabolites of *S. rimosus* and *A. terreus*, namely, oxytetracycline and mevinolinic acid, were determined by employing analytical standards.

The microscopic observations were conducted by using an OLYMPUS BX53 light microscope equipped with an OLYMPUS DP27 high-resolution RGB camera and OLYMPUS cellSens Dimension Desktop 1.16 software (Olympus Corporation, Tokyo).

### Statistical analysis

The results from three experiments were presented as the mean values ± standard deviations (n = 3). For the secondary metabolites that were detected both in the cocultures and axenic variants, two-sample t tests (significance level α = 0.05) were used to determine whether the results obtained for a given coculture differed significantly from the corresponding results for the axenic variant. The same type of test was employed for the products found only in coculture variants to determine if the result in the “10:10” coculture (initialized by using equal volumes of *S. rimosus* and *A. terreus* precultures) differed significantly from the results recorded for a given coculture. All calculations were performed in OriginPro 2017 (OriginLab Corporation, USA).

## Results

### The repertoire of secondary metabolites in “*A. terreus* vs. *S. rimosus*” cocultures

The cocultures of *A. terreus* and *S. rimosus* were initiated by applying 23 different inoculation volume ratios. For comparative purposes, the axenic cultures were generated in parallel with the cocultures. In total, 15 secondary metabolites were detected via UPLC-MS analysis over the course of the study (Table 2). Their identities were either confirmed with the use of reference standards or, whenever the standards were unavailable, assigned putatively on the basis of *m/z* values and literature records.

**Table 2:** The secondary metabolites identified in the axenic cultures and cocultures of *A. terreus* and *S. rimosus*

Retention time (min)	Found *m/z* (ESI −)	Suggested formula	Calculated *m/z* (ESI −)	Δ(*m/z*) = found *m/z* − calculated *m/z*	Suggested metabolite	Level of identification	Producer	Literature
4.4	459.1427	C_22_H_23_N_2_O_9_	459.1404	+ 0.0023	Oxytetracycline	Identified (confirmed with standard)	*S. rimosus*	Stephens et al. (1952)
4.8	458.1409	C_23_H_24_NO_9_	458.1451	− 0.0042	2-Acetyl-2-decarboxamido-oxytetracycline (ADOTC)	Putative annotation	*S. rimosus*	Hochstein et al. (1960)
8.0	766.3990	C_39_H_60_NO_14_	766.4014	− 0.0024	Rimocidin	Putative annotation	*S. rimosus*	Seco et al. (2004)
6.8	738.3635	C_37_H_56_NO_14_	738.3701	− 0.0066	CE-108	Putative annotation	*S. rimosus*	Seco et al. (2004)
7.4	752.3879	C_38_H_58_NO_14_	752.3857	+ 0.0022	Rimocidin—[CH_2_]	Putative annotation	*S. rimosus*	Boruta et al. (2021)
9.0	593.3038	C_31_H_45_O_11_	593.2962	+ 0.0076	Milbemycin β_11_ + [O_4_]	Putative annotation	*S. rimosus*	Boruta et al. (2021)
8.1	720.3937	C_38_H_58_NO_12_	720.3959	− 0.0022	Rimocidin—[CH_2_O_2_]	Putative annotation	*S. rimosus*	Boruta et al. (2021)
12.6	421.2598	C_24_H_37_O_6_	421.2590	+ 0.0008	Mevinolinic acid (β-hydroxy acid form of lovastatin)	Identified (confirmed with standard)	*A. terreus*	Alberts et al. (1980)
13.7	423.2722	C_24_H_39_O_6_	423.2747	− 0.0025	4a,5-Dihydromevinolinic acid	Putative annotation	*A. terreus*	Albers-Schönberg et al. (1981)
8.5	339.2166	C_19_H_31_O_5_	399.2172	− 0.0006	3α-Hydroxy-3,5-dihydromonacolin L	Putative annotation	*A. terreus*	Nakamura et al. (1990)
11.3	396.9890	C_17_H_11_O_7_Cl_2_	396.9882	+ 0.0008	(+)-Geodin	Identified (confirmed with standard)	*A. terreus*	Calam et al. (1947)
9.9	382.9689	C_16_H_9_O_7_Cl_2_	382.9725	− 0.0036	(+)-Erdin	Putative annotation	*A. terreus*	Calam et al. (1947)
11.1	423.1462	C_24_H_23_O_7_	423.1444	+ 0.0018	Butyrolactone I	Identified (confirmed with standard)	*A. terreus*	Rao et al. (2000)
8.6	523.1614	C_28_H_28_O_10_	523.1610	+ 0.0004	4’,8”-Diacetoxy butyrolactone VI	Putative annotation	*A. terreus*	Liu et al. (2018)
9.4	581.1630	C_30_H_30_O_12_	581.1664	− 0.0034	Unknown metabolite	–	–	–

As far as the products originating from *A. terreus* were concerned, the identified molecules represented three biosynthetic pathways, namely, those leading to mevinolinic acid, (+)-geodin, and butyrolactones. Specifically, mevinolinic acid, the main secondary metabolic product of *A. terreus*, was accompanied by two closely related compounds, 4a,5-dihydromevinolinic acid and 3α-hydroxy-3,5-dihydromonacolin L. Furthermore, the metabolite (+)-geodin was identified alongside its structural derivative (+)-erdin. Finally, the presence of butyrolactone I and 4’,8”-diacetoxy butyrolactone VI was also recorded. The actinomycete *S. rimosus* contributed to the coculture biosynthetic repertoire mostly by forming molecules related to oxytetracycline and rimocidin. The former group of compounds included oxytetracycline and its derivative 2-acetyl-2-decarboxamido-oxytetracycline (ADOTC). The latter group included several chemical entities putatively annotated as members of the rimocidin biosynthetic family, i.e., rimocidin, CE-108, and the two rimocidin derivatives formed via the elimination of “CH_2_” or “CH_2_O_2_” groups from rimocidin, hereafter referred to as “rimocidin—[CH_2_]” and “rimocidin – [CH_2_O_2_]”, respectively. In addition, the product previously identified (Boruta et al. 2021) as the oxidized version of milbemycin β_11_, hereafter referred to as “milbemycin β_11_ + [O_4_]”, was detected in the coculture fermentation broth. For one of the detected products, which displayed a *m/z* value of 581.1630 under ESI— ionization (Table 2), the experimental data did not match any secondary metabolites previously described in the context of *A. terreus* or *S. rimosus* cultures.

### The production of secondary metabolites that were identified both in the *A. terreus* axenic culture and “*A. terreus* vs. *S. rimosus*” cocultures

**Fig. 1:**
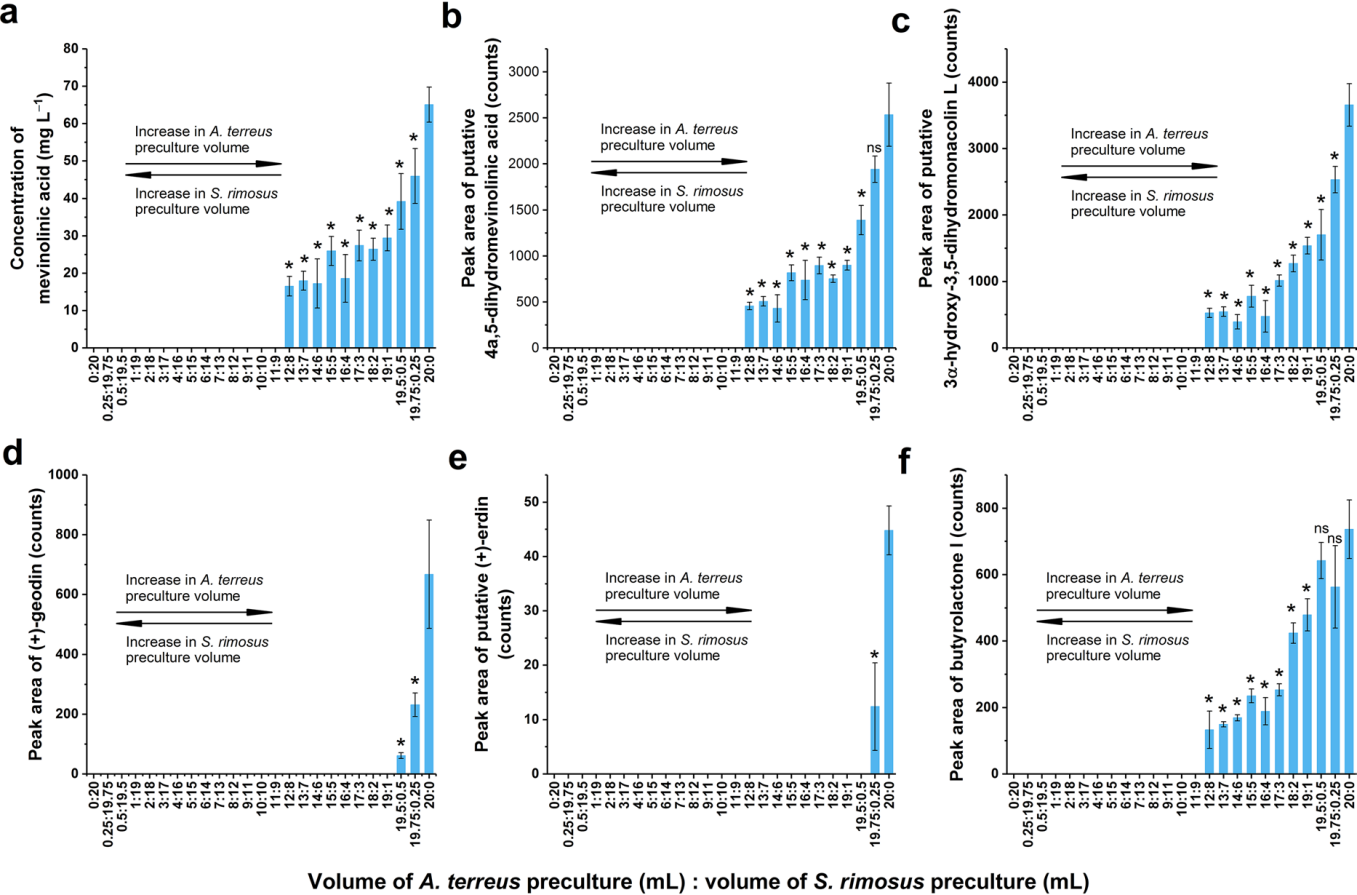
The levels of secondary metabolites identified in the *A. terreus* axenic culture as well as in the “*A. terreus* vs. *S. rimosus*” cocultures, including **a** mevinolinic acid, **b** 4a,5-dihydromevinolinic acid, **c** 3α-hydroxy-3,5-dihydromonacolin L, **d** (+)-geodin, **e** (+)-erdin, and **f** butyrolactone I. The cocultures were initiated by using a range of inoculation volume ratios depicted as the *x*-axis tick labels. The axenic cultures of *S. rimosus* and *A. terreus* were represented by the “0:20” and “20:0” variants, respectively. In this notation, the numbers separated by colons refer to the volumes (in mL) of *A. terreus* and *S. rimosus* precultures used for the initiation of precultures. The concentration of mevinolinic acid was determined by using the analytical standard, while the remaining products were analyzed semiquantitatively by considering the peak area values corresponding to their [M–H]^−^ ions. The identities of mevinolinic acid, (+)-geodin and butyrolactone I were confirmed by referring to commercially available authentic standards, while the remaining metabolites were putatively annotated. The depicted results represent the mean values obtained in 3 experiments, while the error bars correspond to ± standard deviation. The results that differed significantly (P < 0.05) from the values obtained for the *A. terreus* axenic culture (i.e., the “20:0” variant) are marked with “*”, while “ns” denotes the lack of a statistically significant difference

Among the identified metabolites, 6 major products of *A. terreus* were identified both in the axenic variant and the “*A. terreus* vs. *S. rimosus*” cocultures. This group included the molecules originating from the mevinolinic acid pathway, namely, its final products mevinolinic acid (Fig. 1a), 4a,5-dihydromevinolinic acid (Fig. 1b), and 3α-hydroxy-3,5-dihydromonacolin L (Fig. 1c). Their presence was confirmed in cocultures with inoculation volume ratios ranging from 12:8 to 20:0 (Fig. 1a–c), where the first and second numbers represented the volume (in mL) of the *A. terreus* and *S. rimosus* precultures used for inoculation, respectively. In contrast, no traces of these molecules were found when the volume of *A. terreus* inoculum was 11 mL or less (Fig. 1a–c). The same observation was made with regard to the production of butyrolactone I, which was detected solely within the range of inoculation volume ratios from 12:8 to 20:0 (Fig. 1f). However, this behavior was not shared among all the identified fungal metabolites.

For (+)-geodin (Fig. 1d) and (+)-erdin (Fig. 1e), two molecules representing the octaketide biosynthetic pathway in *A. terreus*, the inoculation ratio range under which their production occurred was much narrower than that for mevinolinic acid-related metabolites or butyrolactone I. Specifically, a volume of *S. rimosus* preculture as small as 1 mL led to the complete shutdown of (+)-geodin biosynthesis (Fig. 1d), while in the case of (+)-erdin, even a smaller volume of 0.5 mL resulted in the same blocking effect (Fig. 1e). Therefore, the results clearly indicated that the influence of the inoculation volume ratio on the production of major *A. terreus* products in cocultures with *S. rimosus* was dependent upon the biosynthetic origin of secondary metabolites. Notably, the coculture initiation method involving equal volumes of *A. terreus* and *S. rimosus* precultures (i.e., the “10:10” variant) resulted in a lack of *A. terreus* metabolites in the axenic culture.

### The production of secondary metabolites that were identified both in the *S. rimosus* axenic culture and “*A. terreus* vs. *S. rimosus*” cocultures

**Fig. 2:**
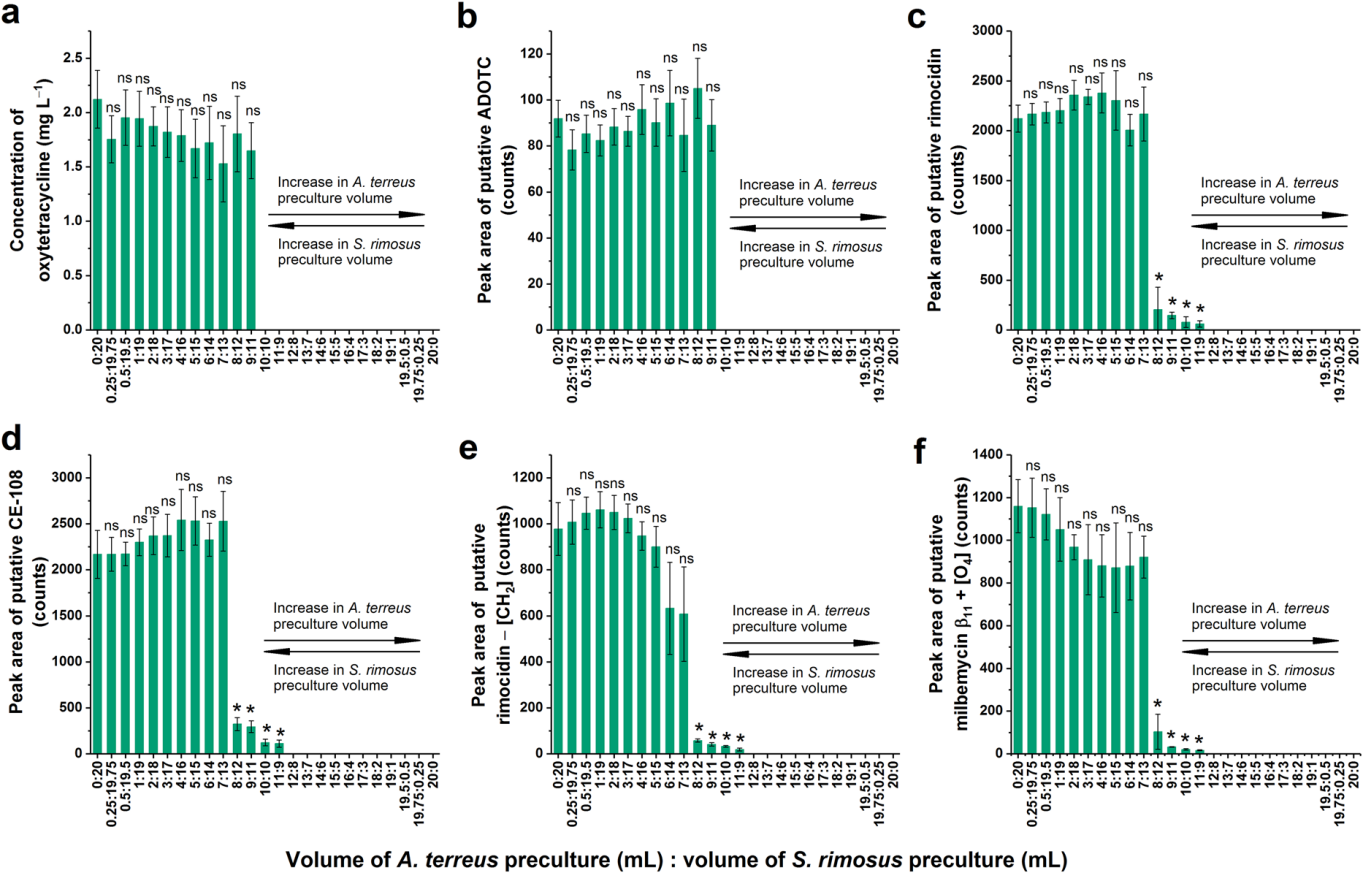
The levels of secondary metabolites identified in the *S. rimosus* axenic culture as well as in the “*A. terreus* vs. *S. rimosus*” cocultures, including **a** oxytetracycline, **b** 2-acetyl-2-decarboxamido-oxytetracycline (ADOTC), **c** rimocidin, **d** CE-108, **e** rimocidin − [CH_2_], and **f** milbemycin β_11_ + [O_4_]. The cocultures were initiated by using a range of inoculation volume ratios depicted as the *x*-axis tick labels. The axenic cultures of *S. rimosus* and *A. terreus* were represented by the “0:20” and “20:0” variants, respectively. In this notation, the numbers separated by colons refer to the volumes (in mL) of *A. terreus* and *S. rimosus* precultures used for the initiation of precultures. The identity of oxytetracycline was confirmed by referring to a commercially available authentic standard, while the remaining metabolites were putatively annotated. The concentration of oxytetracycline was determined by using the analytical standard, while the remaining products were analyzed semiquantitatively by considering the peak area values corresponding to their [M–H]^−^ ions. The depicted results represent the mean values obtained in 3 experiments, while the error bars correspond to ± standard deviation. The results that differed significantly (P < 0.05) from the values obtained for the *S. rimosus* axenic culture (i.e., the “0:20” variant) are marked with “*”, while “ns” denotes the lack of a statistically significant difference

The analysis revealed that 6 secondary metabolites that were produced both in the axenic cultures of *S. rimosus* and the “*A. terreus* vs. *S. rimosus*” cocultures, which included oxytetracycline (Fig. 2a), ADOTC (Fig. 2b), rimocidin (Fig. 2c), CE-108 (Fig. 2d), “rimocidin − [CH_2_]” (Fig. 2e), and “milbemycin β_11_ + [O_4_]” (Fig. 2f).

The production of oxytetracycline (Fig. 2a) and its derivative ADOTC (Fig. 2b) was not recorded in the “10:10” variant. However, their presence was revealed in cocultures for which at least 11 mL of *S. rimosus* preculture was used for inoculation. Markedly different results were observed for the rimocidins (Fig. 2c–e) and the “milbemycin β_11_ + [O_4_]” molecule (Fig. 2f). When the inoculation ratio was within the range from 0:20 to 7:13, these metabolites were produced at levels comparable to those recorded in the *S. rimosus* axenic variant. Within the range from 8:12 to 11:9, their levels were visibly lower, but production was still confirmed. Finally, no traces of rimocidins or milbemycins were detected in the variants corresponding to the inoculation ratios ranging from 12:8 to 20:0 (Fig. 2c–f).

When the production of *S. rimosus* metabolites (Fig. 2) was compared with the results obtained for the *A. terreus* products (Fig. 1), differences between these two datasets were evident. Firstly, no *A. terreus* metabolites were detected at an inoculation ratio of 10:10, while the production of several *S. rimosus* products was confirmed in this variant (albeit at low levels). Secondly, in the cocultures showing nonzero levels of *A. terreus* metabolites, the quantities of these molecules were significantly lower than those in the *A. terreus* axenic culture (Fig. 1). Such behavior was not recorded with regard to the *S. rimosus* metabolites formed in cocultures. For the majority of inoculation ratio variants that yielded nonzero production, the levels of *S. rimosus* metabolites were comparable to the quantities found in the axenic culture (Fig. 2). Despite the differences between the *A. terreus* and *S. rimosus* datasets, there were also similarities that deserve to be mentioned. Notably, none of the industrially relevant secondary metabolites found in the axenic cultures, namely, mevinolinic acid and oxytetracycline, could be detected in the 10:10 variant. In addition, both datasets provided evidence that the influence of the inoculation volume ratio was not universally maintained across the entire secondary metabolic spectrum of a given species.

### The production of secondary metabolites that were identified only in “*A. terreus* vs. *S. rimosus*” cocultures

**Fig. 3:**
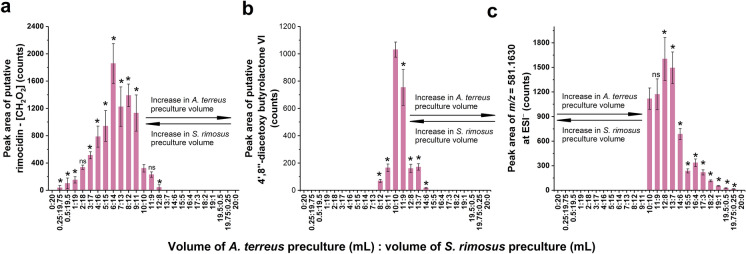
The levels of secondary metabolites identified exclusively in the “*A. terreus* vs. *S. rimosus*” cocultures (i.e., absent from the axenic cultures of *A. terreus* and *S. rimosus*), including **a** rimocidin—[CH_2_O_2_], **b** 4',8''-diacetoxy butyrolactone VI, and **c** an unknown metabolite of *m/z* = 581.1630. The cocultures were initiated by using a range of inoculation volume ratios depicted as the *x*-axis tick labels. The axenic cultures of *S. rimosus* and *A. terreus* were represented by the “0:20” and “20:0” variants, respectively. In this notation, the numbers separated by colons refer to the volumes (in mL) of *A. terreus* and *S. rimosus* precultures used for the initiation of precultures. All three products were putatively annotated and analyzed semiquantitatively by considering the peak area values corresponding to their [M–H]^−^ ions. The depicted results represent the mean values obtained in 3 experiments, while the error bars correspond to ± standard deviation. The results that differ significantly (P < 0.05) from the values obtained for the coculture inoculated with equal volumes of *A. terreus* and *S. rimosus* precultures (i.e., the “10:10” variant) are marked with “*”, while “ns” denotes the lack of a statistically significant difference

In contrast to the secondary metabolites that were found both in the axenic and coculture variants, 3 products found solely in cocultures were revealed over the course of UPLC-MS analysis, namely, “rimocidin—[CH_2_O_2_]” (Fig. 3a), 4’,8”-diacetoxy butyrolactone VI (Fig. 3b), and a metabolite of unknown identity (Fig. 3c). They shared a common characteristic, namely, they were all absent in the “20:0” and “0:20” variants but present in the “10:10” coculture (Fig. 3).

With respect to the levels of these metabolites, each molecule exhibited a distinct type of behavior. The highest levels of “rimocidin—[CH_2_O_2_]” were recorded when the volume of *S. rimosus* preculture used for inoculation was greater than the volume of *A. terreus* preculture. Technically, if the x-axis in Fig. 3 was used as a reference, the variants that favored “rimocidin—[CH_2_O_2_]” biosynthesis were situated to the left of the “10:10” coculture along the axis (Fig. 3a). The highest mean peak areas were recorded when the volumes of the *A. terreus* and *S. rimosus* precultures were 6 and 14 mL, respectively. Markedly different observations were made with regard to 4’,8”-diacetoxy butyrolactone VI. In this case, the most effective stimulation of the biosynthetic pathway occurred when equal volumes of *A. terreus* and *S. rimosus* precultures were applied for coculture initiation, i.e., for the 10:10 variant (Fig. 3b). Finally, the third metabolite found exclusively under cocultivation conditions (Fig. 3c) was detected at the highest levels in the variants for which the volume of *A. terreus* preculture was greater than the volume of *S. rimosus* preculture. If, once more, the x-axis of Fig. 3 is used as a reference, the most favorable conditions for the production of this metabolite were found in the variants situated to the right of the “10:10” coculture (Fig. 3c). Moreover, the inoculation with 12 mL and 8 mL of *A. terreus* and *S. rimosus* precultures, respectively, led to the highest mean peak area among the tested inoculation ratios. To sum up, it was advantageous for the production of “rimocidin—[CH_2_O_2_]” to use an excess of *S. rimosus* preculture relative to *A. terreus* preculture, whereas the reverse recommendation was valid for the unknown metabolite. The effect of inoculation ratio on the biosynthesis of 4',8''-diacetoxy butyrolactone VI was situated somewhere between these two, as the inoculation with equal volumes of *A. terreus* and *S. rimosus* resulted in the enhanced production in this case.

### The morphological perspective on the production of secondary metabolites

**Fig. 4:**
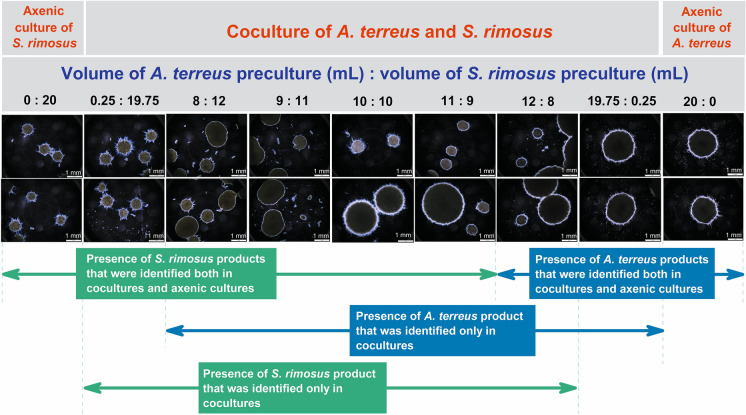
The inoculation ratio range associated with secondary metabolite production is shown in relation to morphological forms observed in submerged cocultures and axenic cultures of *A. terreus* and *S. rimosus*. For clarity, only the selected experimental variants are depicted in the figure

In addition to the secondary metabolite analysis, the visual assessment of filamentous morphologies was performed by employing light microscopy. The pellets of *S. rimosus* and *A. terreus* formed in the axenic cultures were easily distinguishable, primarily due to the difference in size, i.e., the pellets of *A. terreus* were visibly larger than the pellets of *S. rimosus* (Fig. 4).

According to the analysis, the changes in terms of the inoculation volume ratio led not only to metabolic but also to morphological differences among some of the tested variants. Increasing the volume of the *A. terreus* preculture from 0 to 7 mL resulted in practically no change in morphology, as these cocultures strongly resembled the axenic culture of *S. rimosus*. The pelleted forms of similar size and shape, which did not display noticeable differences compared to *S. rimosus* pellets, were recorded for these cocultures.

As the volume of fungal preculture increased relative to the volume of actinomycete inoculum, the morphological characteristics also changed. In the variant initiated by using 8 and 12 mL of *A. terreus* and *S. rimosus* precultures, respectively, the larger pellets that resembled the morphological forms recorded in the *A. terreus* axenic culture were noticeable next to the smaller *S. rimosus*-like pellets (Fig. 4). This observation remained valid for the “9:11” variant; however, in the cocultures ranging from “10:10: to “19.75:0.25”, the outer regions of the larger pellets seemed more hairy than in the “8:12” and “9:11” variants. For the cocultures started by using a volume of *A. terreus* preculture greater than 12 mL, the smaller *S. rimosus*-like pellets were no longer noticeable, and the larger *A. terreus*-like pellets were the only morphological forms present in the fermentation broth (Fig. 4). In summary, the *S. rimosus*-like and *A. terreus*-like pellets were both visible in the coculture, provided that the volume of the *A. terreus* preculture was not greater than 12 mL and was not smaller than 8 mL. In other words, two distinct morphological forms were recorded for the coculture variants with the inoculation ratio ranging from 8:12 to 12:8.

Notably, correlations between the production-related performance and the morphological characteristics of the cocultures were detected. Generally, the presence of *S. rimosus* secondary metabolites corresponded with the presence of *S. rimosus*-like pellets, i.e., in the variants for which the volume of *A. terreus* preculture did not exceed 12 mL. Analogously, the products of *A. terreus* were detectable in the coculture broth provided that the volume of the *A. terreus* preculture was not less than 8 mL (Fig. 4). Interestingly, the range of inoculation ratios that promoted the formation of products from both the axenic cultures and the cocultures (e.g., mevinolinic acid, butyrolactone I or oxytetracycline) differed from the range corresponding to the coculture-specific secondary metabolites (i.e., “rimocidin—[CH_2_O_2_]”, 4’,8”-diacetoxy butyrolactone VI, and the unknown metabolite). With regard to the former group of molecules, the presence of *S. rimosus* metabolites occurred in the variants for which the volume of *A. terreus* preculture was not greater than 11 mL, while the biosynthesis of *A. terreus* metabolites occurred at a fungal preculture volume of 12 mL or greater. Compared with the molecules found both in the axenic and coculture variants, the production of a coculture-specific metabolite originating from *A. terreus* required a greater proportion of the *S. rimosus* preculture to be blocked, as schematically depicted in Fig. 4. Similarly, the formation of a coculture-specific product of *S. rimosus* was still recorded for the “12:8” variant, which did not support the biosynthesis of oxytretracycline, rimocidin or other major metabolites commonly produced by this species.

## Discussion

In axenic cultures, the inoculated microorganism develops without being influenced by any other species. In the case of “*A. terreus* vs. *S. rimosus*” coculture; however, what emerges from experimental work is a picture of two competing microbial rivals. The term “microbial war” might be suitable for describing the interactions between these two filamentous microorganisms (Boruta et al. 2021), even though one may argue, and rightfully so, that such a point view is in fact an oversimplification of a complex biological system. The present study demonstrated that the outcomes of cocultivation are largely shaped by the initial conditions of the bioprocess. Most importantly, the inoculation ratio is among the factors that determine the repertoire of secondary metabolites and their relative quantities.

The current experiment clearly demonstrated the relationship between the inoculation ratio and the recorded production levels. It should be emphasized that this relationship was dependent on whether the given molecule was formed exclusively in cocultures or rather observed both in the axenic and coculture variants. In the former case, the highest mean production levels were observed at the *A. terreus*-to-*S. rimosus* preculture proportions within the range from 6:14 to 12:8, and the levels decreased as the inoculation ratio tended toward the axenic conditions (Fig. 3). These results suggested that the overgrowth of one species by the accompanying microorganism was detrimental to the production of such molecules and that the biomass of both species was comparably important for promoting the corresponding biosynthetic pathways. If the difference in terms of preculture volumes was relatively large, as in the “1:19” or “19:1” variants, the levels of the coculture-specific metabolites were either very low (Fig. 3a,c) or equal to zero (Fig. 3b). Another factor that affected the production profile of a given metabolite was its microbial origin. With regard to the major products of *A. terreus*, such as mevinolinic acid or butyrolactone I, the levels recorded for the cocultures were in almost all cases lower than those in the axenic culture. Generally, the greater the content of *A. terreus* preculture in the inoculum was, the greater the mean concentration or peak area values achieved, reaching their maximum in the axenic cultures (Fig. 1). The situation was markedly different for the commonly observed metabolic products of *S. rimosus*, e.g., oxytetracycline or rimocidin (Fig. 2). At a certain inoculation ratio, these metabolites did not significantly decrease in production with increasing *A. terreus* preculture volume. When the proportion of *A. terreus* relative to *S. rimosus* preculture was high enough, their biosynthesis was either shut down, as in the cases of oxytetracycline (Fig. 2a) and ADOTC (Fig. 2b), or decreased to very small, albeit still detectable, levels, as recorded for the rimocidins (Fig. 2c,d,e) and the derivative of milbemycin (Fig. 2f). Hence, increasing the presence of *A. terreus* in the inoculation mixture failed to negatively affect the production of *S. rimosus* up to a certain critical value of the inoculation ratio (Fig. 2), whereas in the case of most *A. terreus* metabolites, even a small volume of *S. rimosus* preculture (i.e., as small as 0.25 mL) resulted in observable inhibitory effects compared with those of the axenic culture (Fig. 1). Hence, it may be argued that *S. rimosus* inhibited *A. terreus* more strongly than *A. terreus* inhibited *S. rimosus*. There are several factors that might have contributed to this effect, including different growth rates of the actinomycete and the fungus, the production of bioactive secondary metabolites that affected the accompanying microbe, and the mechanisms of morphological development. While it was not possible to determine the contribution of these factors, the outcome of cocultivation could be seen as a net effect of all of them. Moreover, one cannot exclude the role played by other physical or chemical interactions in the cocultivation system that are not yet understood.

The cocultures were all inoculated by introducing the 24-h precultures into sterile production medium. After 24 h of cultivation, the *S. rimosus* and *A. terreus* precultures contained biomass in the form of pellets, which were then used to start the cocultures. However, after 168 h of cocultivation, the *S. rimosus*-like (smaller) and *A. terreus*-like (larger) pellets were simultaneously recorded only within the inoculation ratio range from 8:12 to 12:8 (Fig. 4). Outside this range, one could find only the former or the latter morphological structures, but they were never observed simultaneously in a given coculture variant. Considering previously published observations (Boruta et al. 2019), the likely scenario that occurred here was the engulfment of the pellets of the “weaker” species by the biomass of the accompanying microbe, which was dominant under a given inoculation ratio. In the cases involving relatively large volumes of *S. rimosus* preculture (i.e., from 11 to 19.75 mL), it was the actinomycete that morphologically dominated the cocultures, thus, the larger *A. terreus*-like pellets were absent from these variants. Similarly, a volume of *A. terreus* preculture ranging from 11 to 19.75 mL resulted in the morphological dominance of the fungus and the elimination of smaller *S. rimosus*-like pelleted structures (Fig. 4). It should be noted, however, that the production of a given secondary metabolite in cocultures, as well as in axenic cultures, cannot be attributed merely to the growth of the producing strain. Moreover, the biosynthetic pathways responsible for the formation of these molecules are unique in terms of the environmental stimuli to which they respond. The present study perfectly illustrated these facts. The metabolites of *A. terreus* were produced within distinct inoculation ratio ranges (Fig. 1), and the same observation held true for *S. rimosus* products (Fig. 2).

The absence of oxytetracycline and very low levels of rimocidin in the “10:10” coculture variant (Fig. 2a,c) were not expected, because in a previous study on “*A. terreus* vs. *S. rimosus*” cocultivation (which was entirely based on the inoculation method involving equal volumes of fungal and actinomycete precultures) the levels of these metabolites reached in the cocultures were typically comparable to those recorded for their axenic counterparts (Boruta et al. 2021). However, the cultivation conditions employed by Boruta et al. (2021) differed greatly from those used in the present study. Most importantly, shake flask cultivation (with a total flask volume of 500 mL) was employed here, whereas Boruta et al. (2021) propagated the cocultures in a 5-L stirred tank bioreactor. It can be assumed, based on the common knowledge of bioprocess engineering, that the hydrodynamic conditions and oxygen transfer in these two cultivation systems were not equivalent. Another difference between the two studies was associated with the volumes of precultures used for coculture initiation. Here, a total inoculum volume of 20 mL was introduced into sterile medium to obtain an initial coculture volume of 220 mL, whereas in a previous study 600 mL of inoculum was used to reach an initial coculture volume of 5.5 L (Boruta et al. 2021). Additionally, the two studies differed with respect to the chosen medium composition. Overall, the discrepancies between the two studies exemplified the fact that the production of secondary metabolites in microbial cocultures depends on multiple bioprocess-related factors that require careful consideration and should be taken into account when designing and investigating cocultivation systems. However, the dependency of biosynthetic activities on a wide spectrum of environmental factors is a general feature of microbial secondary metabolism (Brakhage 2013), regardless of whether axenic cultures or cocultures are considered.

The following conclusions were drawn upon the results of the study:

- The repertoire and quantity of secondary metabolites in submerged cocultures of *A. terreus* and *S. rimosus* are visibly dependent on the inoculation ratio.
- The inoculation ratio range that supports the biosynthesis of a given secondary metabolite cannot be predicted solely via microscopic observations, i.e., by detecting the pelleted structures resembling typical morphological forms of a given species. For example, the production of mevinolinic acid in the “10:10” variant did not occur, even though the pellets of *A. terreus* were evidently present in the fermentation broth.
- As far as the major secondary metabolites of *A. terreus* and *S. rimosus* are concerned, including mevinolinic acid and oxytetracycline, respectively, the modification of the inoculation ratio does not lead to observable improvements in production relative to that of the corresponding axenic cultures. However, this strategy is effective in terms of awakening and enhancing the production of secondary metabolites that are not biosynthesized under axenic conditions.

## Data Availability

The datasets used and/or analyzed during the current study are available from the corresponding author on reasonable request.
